# Positioning in 5G and 6G Networks—A Survey

**DOI:** 10.3390/s22134757

**Published:** 2022-06-23

**Authors:** Ferenc Mogyorósi, Péter Revisnyei, Azra Pašić, Zsófia Papp, István Törös, Pál Varga, Alija Pašić

**Affiliations:** 1 Department of Telecommunications and Media Informatics, Budapest University of Technology and Economics, Műegyetem rkp. 3, H-1111 Budapest, Hungary; mogyorosi@tmit.bme.hu (F.M.); revisnyei@tmit.bme.hu (P.R.); pasic.azra@edu.bme.hu (A.P.); pasic@tmit.bme.hu (A.P.); 2 Ericsson Research, H-1117 Budapest, Hungary; zsofia.papp@ericsson.com (Z.P.); istvan.toros@ericsson.com (I.T.)

**Keywords:** positioning techniques, machine learning, 5G, 6G, network-based positioning, indoor positioning, asset tracking, positioning use cases

## Abstract

Determining the position of ourselves or our assets has always been important to humans. Technology has helped us, from sextants to outdoor global positioning systems, but real-time indoor positioning has been a challenge. Among the various solutions, network-based positioning became an option with the arrival of 5G mobile networks. The new radio technologies, minimized end-to-end latency, specialized control protocols, and booming computation capacities at the network edge offered the opportunity to leverage the overall capabilities of the 5G network for positioning—indoors and outdoors. This paper provides an overview of network-based positioning, from the basics to advanced, state-of-the-art machine-learning-supported solutions. One of the main contributions is the detailed comparison of machine learning techniques used for network-based positioning. Since new requirements are already in place for 6G networks, our paper makes a leap towards positioning with 6G networks. In order to also highlight the practical side of the topic, application examples from different domains are presented with a special focus on industrial and vehicular scenarios.

## 1. Introduction

Over the past decade, the rapid development and proliferation of the Internet of Things (IoT), cloud computing, and intelligent terminals has led to the application of Location-Based Services (LBS) attracting considerable attention from both academia and industry [1]. In the outdoor environment, satellite-based positioning technologies can provide convenient location services for people to support applications such as vehicle navigation and cargo tracking.

However, indoors and in dense urban areas, the accuracy of satellite-based positioning technologies decreases due to severe occlusion by objects and multipath effects in signal propagation, and cannot meet the requirements of the applications; therefore, there have been continuous efforts in recent decades to find equally (or more) scalable and accurate positioning techniques [2,3,4]. There are several publications that summarize the results and limitations of the various areas of non-satellite-based (or non-GPS) positioning systems, e.g., Visible Light Communication (VLC), Sound, Radio Frequency (RF), or Passive Information (magnetic field or passive sound) based technologies [1,2,3,4,5,6,7,8,9,10,11,12,13,14,15,16,17]. As mobile networking reached global land coverage, the idea of network-based positioning was (literally) in the air. It has become an important use case for 5G as it fits the requirements of scenarios related to vehicles and cyber–physical systems. Increased positioning accuracy also remains a focus for 6G mobile networks.

Because of this importance, there have been several studies that have looked at the positioning capabilities of 5G networks. Most of these works were conducted before commercial 5G networks were widely deployed, so many more recent works are missing. In addition, each study has its own perspective with a fairly specific focus. As a result, there are technologies and methodologies that require more attention. In [15], cellular positioning methods from previous to current standards, their reported performance, and localization perspectives in next-generation cellular networks are presented. The authors of [18] provide a comprehensive overview of the positioning architectures in previous generations of cellular networks and then propose a general positioning architecture for 5G networks, leveraging the new features of emerging technologies. The technology and methods of millimeter Wave (mmWave) and massive Multiple-Input Multiple-Output (MIMO) localization in 5G networks are discussed in [19,20]. Cooperative positioning technologies and methods are presented in [21]. The security and privacy threats according to the participants of 5G positioning are presented in [22]. An overview of 5G positioning solutions in smart cities is given in [23]. Ref. [24] presents a study on integrated localization and communication (ILAC) in 6G networks. Ref. [25] discusses the technical trends and opportunities in 6G networks to achieve latency and accuracy improvements and low-cost positioning.

The main goal of this paper is to provide a comprehensive overview of machine-learning-aided and traditional cellular-based positioning techniques and architectures in 5G and 6G networks. To do this comprehensively, we have provided a brief summary on positioning in cellular networks. Furthermore, we surveyed the conventional positioning solutions (which do not use machine learning) for 5G networks, as such overviews were still lacking—in particular, the comparison of the advances in the field. At the end of the chapters on conventional 5G-based positioning and machine-learning-based positioning, we present our key findings to briefly summarize the main results of these long surveys.

In this paper, we focus on the machine-learning-aided cellular-based positioning techniques and architectures utilizing the improvements of 5G networks. The contributions of this paper are the following:

- Section 2 is an overview of the characteristics of cellular-network-based positioning—the different types of cellular positioning are summarized here.
- Section 3 describes the capabilities of conventional positioning techniques, and several non-ML positioning solutions are introduced and compared here.
- An extensive study of ML-aided positioning techniques is provided in Section 4, and the different methods are compared to each other based on positioning accuracy.
- Section 5 summarizes the expected advancements of 6G networks in terms of positioning.
- Major results of real-world use cases that have been published within the scientific communities so far are collected in Section 6.

At the end of the paper, Section 7 concludes this research, summarizing the main findings.

## 2. Positioning in Cellular Networks

In this section, we present the most relevant cellular positioning technologies, discuss the applicability of ML technologies to improve their accuracy, and highlight the advances of 5G networks. Accurate and real-time positioning is highly demanded by location-based services and can be beneficial for radio resource management in 5G networks, which are deployed to achieve significant performance improvements over existing cellular networks. Many new technologies, e.g., massive MIMO, mmWave communications, Ultra-Dense Network (UDN), and Device-to-Device (D2D) communications, are being introduced in 5G networks to not only improve communication performance, but also provide the ability to significantly increase positioning accuracy [18]. It is envisioned that 5G networks will be able to locate User Equipment (UE) with sub-meter accuracy and provide high network utility [26], such as seamless coverage, low delay, and high throughput, placing higher requirements for cellular communication systems.

**Figure 1:**
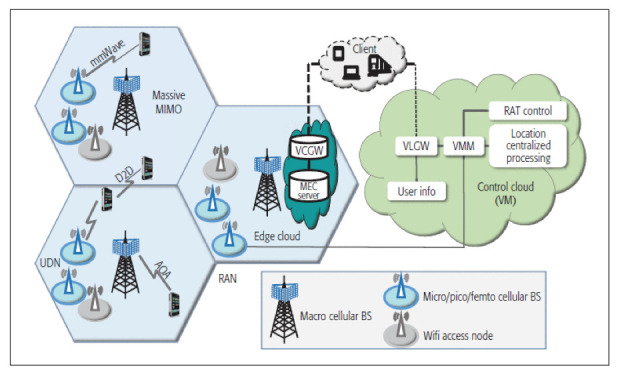
An example for a 5G positioning architecture [18].

In cellular-based localization, the downlink transmissions from the Base Station (BS) to the mobile device and uplink transmissions from the mobile device to BS can be utilized to determine the position of the UE [18]. The cellular-positioning methods can be classified into two main categories depending on the entity that computes the position: (1) mobile-based, where the UE itself calculates its location, and (2) network-based, where the network location server computes the position of the UE. Most cellular-based positioning solutions are network-based due to its centralized nature that allows full control of the location service by the network operator, as well as its support to legacy devices. As shown in Figure 1, the 5G positioning architecture mainly consists of two parts: a Radio Access Network (RAN), which includes a multi-Radio Access Technology (RAT) network, edge cloud, and control cloud, where the location of the UE is determined based on the measurements of the UE and BSs [18]. Every reviewed solution is network-based in this section.

Cellular-based localization technologies can be divided into four main categories: cell-identity-based, angle-based, range-based, and fingerprinting-based [18].

### 2.1. Cell-Identity-Based Localization Techniques

The Cell identity (CID) or proximity-based technique is the simplest of the four techniques, as it mainly relies on checking whether or not the object to be positioned is present in a particular radio coverage area. It is necessary to know the location of the serving base station and the area of the serving cell that is incorporated to estimate the UE’s location. It requires many base stations to achieve comparable accuracy to other methods and is not suited for large areas or areas with low-density populations [27].

### 2.2. Angle-Based Localization Techniques

**Figure 2:**
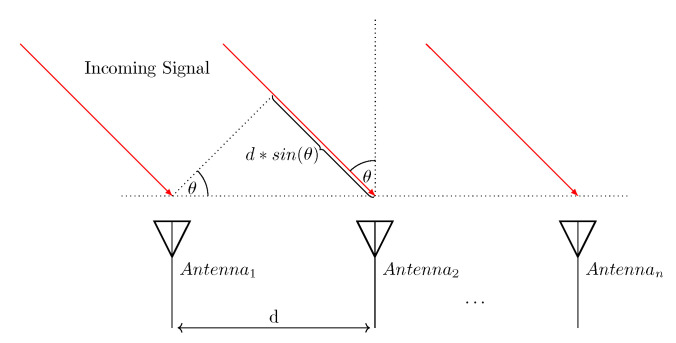
The angle of arrival (θ) can be calculated with the measurement of the incoming signal’s phase shift between the antennas.

The angle-based techniques utilize either the Angle-of-Arrival (AoA), the Angle-of-Departure (AoD), or even both. The arrival angle is the direction from which the radio signal is received (see Figure 2), while the angle of departure is the direction where the signal is transmitted. In 5G networks, with massive MIMO, the BS will be equipped with hundreds or even thousands of antennas, which can provide an extensive array aperture and support beam-based operations [10]. During the beam search operation, the angle can be obtained easily in terms of the downlink Angle of Departure (DL-AoD) and the uplink Angle of Arrival (UL-AoA). Although UEs and BS search for the best beam for the radio link, DL and UL Reference Signal Received Power (RSRP) is measured to evaluate the signal quality of a beam. This enables the BS to analyze the uplink channels using pilot signals sent by the UEs, which can estimate the AoAs with high accuracy and low inter-user interference effect; therefore, massive MIMO will boost the utilization of angle-based positioning approaches in 5G networks, which are not widely used in 2G–4G networks [18].

Unfortunately, most of the time, the positioning requests are coming from dense multipath environments. In these circumstances, appropriate processing of measurements is essential for accurate positioning since only the Line-Of-Sight (LOS) measurements reflect the real angular relationship between the UE and the BS. This is why the most typical positioning approach is the two-step approach, where first an LOS/Non-Line-Of-Sight (NLOS) identification is performed, and then the identified LOS measurements are used for positioning [18]. In some cases, an LOS path may not even exist in the multipath environment, usually caused by large blocking objects, such as walls. When all signals are NLOS propagated, the localization errors are substantial.

### 2.3. Range-Based Localization Techniques

**Figure 3:**
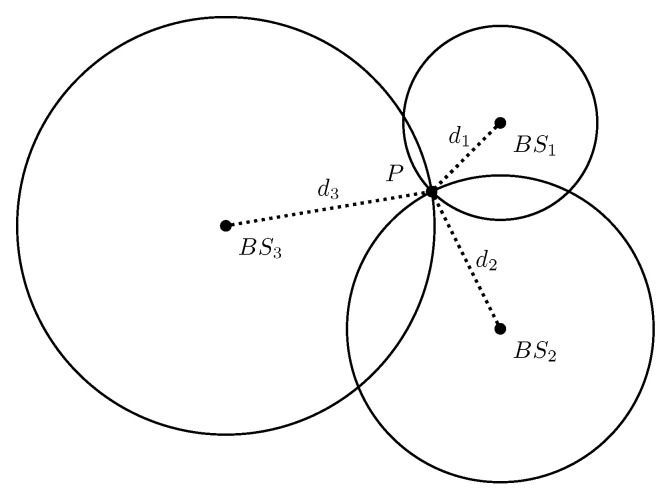
Range measurement based positioning with at least three BS.

**Figure 4:**
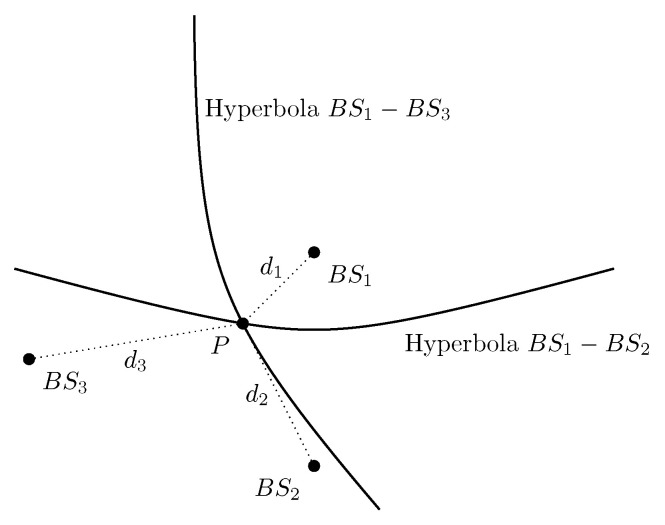
Range difference measurement based positioning with at least three BS.

The range-based localization techniques estimate the unknown position of the UE based on range measurements between transmitters and a receiver (or vice versa). These measurements can be obtained by extracting the information contained in the received signal, such as Received Signal Strength (RSS), Time of Arrival (ToA), and Time Difference of Arrival (TDoA) [28]. As shown in Figure 3 and Figure 4, the distance between the UE and a minimum of three base stations (or one base station if the BS antennas are separated wide enough) is required to estimate the BS location by using trilateration [27].

If there is a misalignment between the user receiver clock and the synchronized transmitter clocks, a common offset (bias) corrupts the measurements, resulting in pseudoranges (a range estimation containing an unknown error). In addition to the unknown user position, this offset needs to be treated as an unknown parameter too. It can be either estimated using the pseudoranges or eliminated by taking differences among the pseudoranges (hence, TDoA). For fully synchronized (ToA) or non-timing-based systems (such as RSS), this type of offset does not exist [28].

The 3GPP NR system supports two types of TDoA measurements: (a) the Observed Time Difference of Arrival (OTDoA), also called downlink-TDoA (DL-TDoA), where the ToA is measured in the downlink; (b) uplink-TDoA (UTDoA), based on sounding reference signals (SRS) [29]. One advantage of UTDoA compared to OTDoA is the time-stamping accuracy, which affects the time measurement accuracy. Time-stamping accuracy depends on the clock frequency and clock drift in the hardware. In the case of OTDoA, the ToA is measured at the UE, which typically gives worse performance than measurements at the BS. Since the UE measurements are performed on separate occasions, the time drift between the measurements becomes an additional error source.

As emphasized in the discussion of the angle-based techniques, in practice, the UE does not have line-of-sight paths to more than one BS, resulting in corrupted ToA measurements. Usually, in non-line-of-sight conditions, the ToA-based solutions have low accuracy.

### 2.4. Fingerprinting-Based Localization Techniques

The most-utilized fingerprinting-based positioning method consists of two phases: an offline (training) phase and an online (positioning) phase. In the offline or training phase, a database is created by measuring given signal or antenna attributes at known locations (i.e., known as signatures or fingerprints). Such a signal attribute can be the received signal strength, which can be measured by the UE. In addition to these attributes, the floor number (if indoors), the orientation of the device, the TA (Timing Advance) or RTT (Round-Trip Time) value (or any kind of timing information), the type of mobile unit, etc., may also be stored. This database of locations and the associated fingerprints are called the radio map or fingerprint database. This database enables the creation of one or more models through Machine Learning (ML) techniques. In the online phase, localization is accomplished by using the model to estimate the user’s position based on the attributes measured by the mobile unit.

The most significant advantage of fingerprinting-based solutions is their ability to remain accurate even in highly cluttered multipath environments [18,30]. It provides an edge over the angle-based and range-based solutions, but it comes with a high cost, too, since the offline phase’s fingerprint collection requires extensive human resources. The Achilles heel of the fingerprinting-based solutions is the alteration of the setup. For example, when the number or the locations of the access points in the environment changes, the radio map, i.e., the fingerprint, must be updated accordingly. Although a simple removal of an access point can be modeled with the deletion of the corresponding values from the fingerprints, the installation of one or more new access points forces a new fingerprint collection. Additionally, the different types of mobile devices contain various types of antennas, resulting in an additional error component in the measurements. These problems negatively affect the accuracy of positioning.

## 3. Conventional Positioning Solutions in 5G Networks

In positioning solutions, the potential of using Machine Learning (ML) models is generally undisputed; however, in some cases, these models are not a viable solution for various reasons (e.g., computational resources, training/testing time, and data quantity and quality). Fortunately, a plethora of 5G positioning methods has already been devised [31] and most of them are capable of achieving high-quality results even without machine learning models; therefore, in this section, we present some of the most prominent solutions without ML for positioning in 5G networks. The experiments to be mentioned are divided into two groups depending on whether 5G is assisted by other technologies or not.

### 3.1. Pure 5G Network-Based Positioning

In this section, we briefly present positioning solutions that require input data based only on 5G signals. As the following solutions show, the data types mentioned in Section 2 can be utilized in various ways for indoor or outdoor positioning.

The reference architectures, signals, and protocol-related peculiarities are well summarized by Dwivedi et al. [32] based on Release 16, as this set of recommendations provides the foundations for 5G positioning. In addition to illustrating the capabilities of the standardized components, they also provide simulation results based on key scenarios with certain assumptions.

Papp et al. [33] investigated the actual realization of a TDoA-based indoor positioning system on existing 5G small cell networks. Their primary focus was on indoor signal propagation and to overcome the challenging effects related to such scenarios. Their approach was to create a channel model based on real 5G measurements and to use the novel model obtained by using that data for the creation of a realistic simulation framework. This framework was then utilized to analyze the performance of various algorithms. They found that using some of these algorithms can significantly reduce the impact of NLOS propagation. Consequently, a positioning error under 3 m is achievable in certain cases. Furthermore, accuracy could be improved not only by increasing the number of ARPs (Antenna Reference Points, radio dots), but further enhancing the RWLS (Robust Weighted Least Squares) method, the power-delay-profile-based (PDP) NLOS algorithms, or by combining TDoA measurements with the results from built-in inertial measurement unit sensors or with the angular measurement data coming from MIMO antenna systems.

Zhang et al. [34] proposed a solution for 3D positioning in a simulated indoor 5G ultra-dense network. In their work, they proposed a 3D dynamic reconstruction fingerprint matching algorithm whose first step is to reconstruct the complete fingerprint matrix from partial data. Then, to simplify the fingerprint data, the suboptimal service base stations are eliminated from the dataset. Finally, the k-nearest neighbor matching algorithm is used to estimate the 3D coordinates. The positioning errors are evaluated at several Signal-to-Noise Ratio (SNR) levels. The mean error at SNR=2 dB is 0.31 m and 0.16 m at SNR=20 dB.

Menta et al. [35] performed their research using a realistic outdoor 5G network testbed. They utilized a two-stage Extended Kalman Filter (EKF) based positioning engine. In the first stage, AoA estimation is performed by local EKF computing engines at each Transmission–Reception Point (TRP). Then, all AoA estimates are fused by a global EKF to obtain the position estimate. According to their results, a sub-meter 2D error is achievable with a probability of 95%. Similarly, Koivisto et al. [36,37] also used a cascaded EKF in a two-stage manner for an unsynchronized 5G ultra-dense network. In their solution, the computations are performed on the network side to minimize the power consumption and computational requirements at the UEs. In the first stage, the ToA and the Direction of Arrival (DoA) of the user nodes are estimated. Then, in the second EKF stage, the individual ToA/DoA estimates are fused to estimate the user node position. Their results show that sub-meter accuracy for positioning and tracking is feasible. The simulations in their work are performed based on the METIS Madrid map model [38]. In their extended research, sub-meter error was also achievable; however, in this case, a realistic clock model with clock skews was used.

Sun et al. [39] focused their experiments on positioning a single cell (base station) with a wideband 5G signal and a vector antenna (VA). Consequently, this solution is free from the complications of multi-cell approaches, such as synchronization between base stations and increased deployment costs due to the complexity of the system. For position estimation, they used statistics-based expectation maximization and the subspace-spaced algorithm. The results based on the sounding reference signals in a line-of-sight scenario prove that VA is capable of providing 3D UE positioning with sub-meter accuracy in 5G networks without requiring multiple cells or multiple antennas. Partly similar to the previous study, Jin et al. [40] conducted their experiments in a virtualized indoor office scenario with only one mmWave Base Station (BS). In their solution, an improved Least Mean Square (LMS) algorithm is used to refine the multipath AoA estimation based on the motion characteristics of the user devices. Then, an Unscented Kalman Filter (UKF) is used to estimate the position based on the AoA and ToA distributions of the different mmWave paths. According to the results, their proposed method is able to achieve a positioning accuracy of less than one meter.

Kakkavas et al. [41] investigated the performance limits of Vehicle-to-Vehicle (V2V) relative positioning for vehicles with multiple antenna arrays. AoA and TDoA measurements are used to estimate the position and direction of the vehicle in platooning and overtaking scenarios. These are used to obtain the Cramer–Rao bound for lateral and longitudinal positioning error. The results are compared to the 5G new radio vehicle-to-everything [42] Positioning Error Bound (PEB) requirements: longitudinal PEB less than 0.5m and lateral PEB less than 1m. In the overtaking scenario, the requirements are met if the longitudinal offset between vehicles is less than 22.46 m. In the platooning scenario, the distance between the vehicles cannot exceed 17.07 m in order to keep the positioning error under the limit value.

### 3.2. Assisted Positioning in 5G Networks

In this section, we present the efficiency of 5G hybrid solutions. The following examples show that GNSS assisted 5G positioning is currently being extensively studied by multiple research groups. In addition, a promising solution is also reviewed, which utilizes 5G signals assisted by visible light communication.

**Figure 5:**
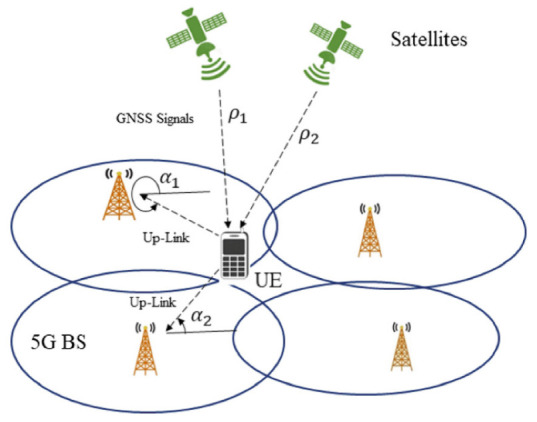
Illustration of the hybrid GNSS-5G positioning [43].

Sun et al. [43] proposed a hybrid 5G-GNSS positioning method (see Figure 5) based on combining AOA estimates from 5G base stations and TOA measurements from GNSS satellites. In their simulations, three satellites are visible. The mathematical models for the GNSS and 5G measurements are nonlinear due to the clock bias between the UE and GNSS system time; therefore, the Taylor series least-square method is used to linearize the mathematical model and estimate the position solution iteratively. Then, moving averaging is performed for the raw position estimates to reduce the effects of noise. According to their results, the 2D position error is less than 10m in more than 95% of the cases with the hybrid 5G-GNSS solution. Under the same circumstances, this value is about 15 m for the standalone 5G AOA positioning method. Thus, by using both GNSS and 5G signals, the authors were able to reduce the positioning error by at least 5 m.

Abu et al. [44] proposed their method of hybrid GNSS and 5G positioning for specific scenarios where systems of Autonomous Vehicles (AVs) are equipped with 5G transceivers. They focused on scenarios where GNSS and 5G positioning systems individually do not work well or have too large error. For example, on urban roads, where AVs can only receive GNSS signals for short periods of time due to limited satellite visibility. The results of their simulations show that when the GNSS satellites are well-spaced and two 5G base stations are available, a sub-meter error is achievable with the hybrid solution. Moreover, the sub-meter error is also obtainable when the GNSS satellites are ill-positioned but two 5G base stations are still accessible. Based on that, the authors concluded that the positioning information of the 5G signals is the main component of this hybrid 5G-GNSS positioning solution.

Yin et al. [45] focused on hybrid 5G-GNSS D2D positioning in their study. The experiments were conducted with a dataset consisting of real GNSS trajectory data and simulated 5G D2D measurements. Moreover, the GNSS dataset contains both parts with good GNSS conditions and parts with denied GNSS. To improve the efficiency of measurements for D2D positioning in 5G networks, a novel Crossover Multiple-Way Ranging protocol (CO-MWR) was proposed. In addition, a particle filter was used to estimate the user’s position. They also proposed a state dimension reduction method to prevent particle degeneracy, which would lead to performance degradation. Their results show that their solution can provide accurate estimates even in areas where GNSS is not available. Meanwhile, an RMSE of 3 m or less can be achieved for the entire data set.

In their study, Yang et al. [46] presented a multi-layer network architecture that integrates visible light communication (VLC) and visible light positioning (VLP) in 5G networks. Their simulations compared VLC assisted 5G and WiFi-based positioning solutions in an indoor environment (one room) with 25 VLC AP and 4 RF AP evenly distributed. According to their results, the mean positioning error was 7.19 cm in case of the VLC-5G-based positioning, in contrast to the WiFi fingerprint algorithm, whose mean error was 68.76 cm. Aside from the mentioned sub-meter positioning error, some features of the VLP technology are worth mentioning: due to the already existing light infrastructures, deployment costs are low, and they can be used in electromagnetic-interference-sensitive scenarios, and communication security is ensured since visible light signals cannot penetrate walls.

### 3.3. Lessons Learned

Based on the previously presented papers (summarized in Table 1), it can be said that there are several promising methods for positioning in 5G networks without utilizing machine learning algorithms. According to several simulation results, sub-meter accuracy could be achieved even if only 5G signals are used. Moreover, the same accuracy is obtainable in cases where the 5G signals are assisted with other technologies such as GNSS or VLC.

It should be noted that in most cases, the positioning methods are tested in a fully or partially simulated environment, due to the lack of deployed 5G networks. Simulations tend to underestimate the effects of the physical environment because many of these effects are difficult to estimate and are highly dependent on the particular environment. The models differ greatly in several parameters, such as the number of LoS base stations, the type of simulated signal, and the size of the virtual space; therefore, comparing these positioning solutions based only on their positioning error could be very misleading. To mitigate these problems, most of the works include sensitivity analysis to show the impact of increasing noise on the accuracy of the algorithm. To make simulation results easily comparable in the future, a common and consistent scale or viewpoint must be established. In addition, creating publicly available indoor and outdoor data sets and using them to evaluate the performance of the algorithms would further improve the comparability and reproducibility of the different positioning solutions.

## 4. Machine-Learning-Aided Positioning in 5G Networks

In this section, we present machine-learning-based methods for indoor and outdoor positioning. The experiments used machine learning approaches to enhance the positioning accuracy in 5G networks and can be divided into groups based on the used localization techniques discussed earlier (Table 2).

### 4.1. Advances in Positioning Aided by Machine Learning

The simplicity is the only advantage of the cell-identity-based techniques; therefore, no experiments were investigated from a machine learning perspective. Even though angle-based methods are one of the main winners of the massive MIMO introduced in 5G, the positioning in the LOS scenario does not require an ML approach [47,48]. On the other hand, even if the angle information cannot be used for accurate positioning (e.g., in a NLOS scenario), it can be utilized to enhance the accuracy of other approaches [49,50,51]. In most cases, the AoA/AoD is used as a fingerprint feature, which can seriously decrease the positioning error in an LOS scenario. Most of the machine learning approaches are attempting either to predict the environmental parameters (e.g., LOS/NLOS) or signal parameters [52].

Malmström et al. [49] have shown that knowledge of the antenna is critical for accurate position estimation, and that it is possible to position UE within 10 m with 80% accuracy using an 8×8 antenna array. Their work explores the use of two ML methods—neural network (NN) and random forest (RF)—to estimate the position of UE in an urban area using antenna beam data. The radio measurements used are Beam Reference Signal Received Power (BRSRP) and AoD from a set of beams from an antenna. In order to have different position models for LOS and NLOS conditions, the authors evaluated two Generalized Likelihood Ratio Test (GLRT) detectors to detect NLOS conditions. The first used the difference between the BRSRP measurements as input, whereas the second used the difference between the AoD measurements. The solution based on the BRSRP difference achieved a detection probability of 88%, whereas the solution based on the AoD difference achieved only 76%. The false positive probability was 5% in both cases. In the NLOS cases, the random-forest-based positioning outperformed the NN-based one (8.4 m vs. 9.2 m); however, under LOS conditions, the NN performed much better (2.1 m vs. 8.4 m).

Decurninge et al. [53] reported experimental results on using a learning-based approach to infer the location of a mobile user of a cellular network within a cell for a 5G-type Massive MIMO system. They investigated the extent to which uplink Channel State Information (CSI) can be used for UE positioning. They chose the normalized covariance of the instantaneous uplink propagation channel vector as the input to the learning algorithms, since according to them, the second-order channel statistics capture most of the location-related characteristics of the CSI. They argued that statistical learning is a viable way to deal with the complexity of the relationship between the spatial scattering environment surrounding a user and the CSI measured by the BS. Two ML approaches were considered: a feedforward Neural Network (NN) and a k-Nearest Neighbor algorithm (kNN). The training and testing data were collected on the Huawei 5G testbed of a university campus. The covered area contains mostly Line-Of-Sight (LOS) locations. The CSI data were measured for a single-antenna UE on the uplink of a massive-MIMO system with a 32 dual-polarized antenna array at the BS. To evaluate the sensitivity of the localization error to the number of antennas, simulations were performed with a varying number of BS antennas (*N*) by discarding part of the data. The results show that the localization error is small and exhibits little sensitivity to *N* as long as N≥12 (mean error ≤14 m), while for N≤8 the localization accuracy decreases significantly (mean error ≥18m). Moreover, the kNN approach (k=3) outperformed the NN-based approach in terms of average accuracy (8.16 m vs. 9.6 m).

Orabi et al. [54] evaluated the ability of different neural networks to mitigate multipath effects in 5G downlink signals, in particular to learn multipath-induced errors in code phase estimation of a 5G receiver. The authors studied two types of neural networks, namely Feed-Forward Neural Networks (FFNNs) and Time-Delay Neural Networks (TDNNs). The NNs use inputs from the AutoCorrelation Function (ACF) to learn the errors in the code phase estimate of a conventional Delay-Locked Loop (DLL). A ray-tracing algorithm is used to model the effects of the Non-Line-Of-Sight (NLOS) components on the Line-Of-Sight (LOS) component and to generate training data. They investigated the sensitivity of the out-of-sample error to the number of hidden layers, the number of neurons per layer, the regularization constant, and the time window of the ACF using five-fold cross-validation. Their simulation results showed that both TDNNs and FFNNs offered RMSE reductions of 29.1% and 59.6%, respectively, over a conventional DLL over the test set. The results highlighted the importance of the available time history, which helped the TDNN extract information from the dynamics of the LOS and NLOS signals. In addition, the authors conducted an experiment to test the generalization ability of the TDNN in real environments. The TDNN showed an overall RMSE reduction of 27.1% with a reduction of 38% in the most severe multipath region.

Comiter et al. [55] proposed a data-driven localization approach for narrow beam alignment for mmWave networks. According to real experiments, their methods achieve sub-meter localization accuracy (median squared error) both indoors (0.33–0.37 m) and outdoors (0.76 m). They argue that their domain-specific neural network approach can increase localization accuracy while minimizing the amount of real samples that need to be collected to train the model. Their approach consists of the following elements: the Structured Multilayer Perceptron model (SMLP), which addresses the problem of collinear regions (localization on the line between 2 BS), a quantized loss function that focuses on points with significant error, and a synthetic data generation procedure to limit the amount of measurements required for accurate localization. They confirm with simulations and real experiments that their methods are robust under different noise models, outperform the conventional NN model, meet the latency requirements of 5G networks, and solve the problem of collinear regions.

Klus et al. [56] proposed a densely connected NN-based solution for increasing the positioning accuracy in an urban beamforming-based network scenario with high RSS uncertainty. The authors combined RSS and GNSS localization data to enhance the positioning performance. They executed their experiments in a ray-tracing-based simulation setup using the Madrid grid layout [38] with 7 BS. The positioning performance of the NN model was evaluated with and without the combined localization data on six different RSS uncertainty levels. On the lowest uncertainty level, the utilization of both the GNSS and RSS data led to a 32% error reduction compared to using only the RSS signal. Moreover, in this case, the mean error is 0.74 m. On the highest uncertainty level, the error reduction is 49% and the mean error is 1.75 m with the fusion NN model.

Butt et al. [57,68] used Deep Neural Networks (DNN) and decision tree regression for positioning based on BRSRP data. An outdoor urban environment was simulated, where the base stations had a 16×16 antenna array. With a network-level DNN model (the DNN training was performed with all the data from all the sites at one location server), the average error is approximately 5 m. Additionally, the authors investigated the positioning accuracy by training individual cell-specific DNN models for each cell. In this scenario, the average error can be as low as 1.4 m.

Gante et al. [50] evaluated several NN architectures for Beamformed Fingerprint (BFF) data-based positioning. They also enhanced the positioning with tracking techniques, which leveraged short-term historical data. The BFF is a 2D matrix, where the data sequences along both dimensions carry valuable information. This is why the Convolutional Neural Network (CNN) and the hierarchical CNN were used for the single BFF positioning. For the BFF tracking problem, where the positioning has to be performed based on a sequence of BFFs, the Long Short-Term Memory Networks (LSTM) and Temporal Convolutional Networks (TCN) were considered. In the single BFF positioning, the average error ranges from 4.57 m to 6.17 m in the case of the CNN and from 3.31 m to 5.13 m in the case of the hierarchical CNN with K=64 partitions, subject to the noise level. The average errors in the BFF tracking are reduced thanks to the increased number of datapoints. At lower sequence numbers, the TCN outperformed the LSTM (2.3 m vs. 3 m), but in the case of longer sequences, the gap shrank while the TCN remained reasonable. In low-noise scenarios, the TCN achieved an average error of 1.78 m.

Sun et al. [58] proposed a fingerprint-based single-site localization method for massive Multiple-Input Multiple-Output (MIMO) orthogonal frequency-division multiplexing (OFDM) systems using both the AOA and PDP parameters as fingerprint. In massive MIMO-OFDM systems, fingerprint extraction and matching has high computational complexity due to the high multipath resolution in the angle and delay domains; therefore, they proposed an efficient fingerprint extraction method with a corresponding similarity function and a fingerprint clustering algorithm with a new location estimation method. The fingerprints are extracted from the channel estimation results through Fast Fourier Transform (FFT) and named as the Angle Delay Channel Power Matrix (ADCPM). The authors argue that the ADCPM contains rich multipath information with clear physical interpretation, such as the channel power associated with the specific AOA and TOA. To exploit the information in fingerprints, they proposed a new similarity criterion for fingerprints, the Joint Angle Delay Similarity Coefficient (JADSC), which is used to calculate the distance between two fingerprints. For fingerprint compression, they developed a two-step fingerprint clustering algorithm for database preprocessing, which uses the spatial characteristics of fingerprints to significantly reduce matching operations. Finally, they used the Weighted K-Nearest Neighbor (WKNN) method for accurate location estimation. Their simulation results show that their fingerprint-based method is able to outperform DiSouL and TF-MUSIC in an outdoor scenario. With multiple BSs, their method achieves 90% reliability at 2 m accuracy and with one BS it provides 95% reliability at 3 m accuracy.

Wang et al. [59] proposed a Deep Convolutional Gaussian Process (DCGP) based regression for fingerprinting-based mmWave positioning in an outdoor environment. For the simulations, the authors used an open-source mmWave dataset, which contains beamforming images from 160,801 bidimensional positions [50]. According to their results, the 95th percentile error for a CNN model is 14.289 m and for the DCGP model it is 7.018 m; however, on their hardware, the training time for the DCGP took longer (533 min) than for the CNN model (376 min). They also evaluated the impact of the number of epochs on the mean distance error. In their experiments, the highest number of epochs was 550, and in this case the mean distance error is 4.46 m for the CNN and 2.79 m for the DCGP.

Al-Rashdan et al. [27] evaluated the performance of thirteen ML algorithms employed in conjunction with fingerprint-based MT localization for distributed massive MIMO wireless systems configurations. The fingerprints used RSRP measurements of one BS solely with an 8×8 antenna array from a simulated outdoor environment. The authors investigated the effect of the number of antennas, the inter-element separation distance, the frequency, and the weather. In each case, the kNN achieved the lowest MAE (Mean Absolute Error), followed by the random forest and gradient boosting. In most cases, the SVM and NN achieved the highest MAE. The lowest MAE of the kNN was 3.3 m in the best-case scenario. The frequency and the weather had no significant effect on the error, while the increased number of antennas and the increased inter-element separation distance decreased the average error.

El Boudani et al. [63] proposed the DEep Learning-based co-operaTive Architecture (DELTA) machine learning model implemented on a 3D multi-layered fingerprint radio map. The RSRP measurements were collected in an indoor environment with the following dimensions: 8 m width ×16 m depth ×2.75 m height. The positioning method begins with estimating the 2D position. Then, the output is used recursively to predict the 3D location of a mobile station. The DNN-based positioning has outperformed the kNN and SVM-based methods. The 2D average error of the DNN was only 1.6 m while the kNN’s was over 2.5 m and the SVM’s was over 5 m. The identification of the height translated into a classification problem where three classes were considered. The SVM had a misclassification rate of 66% while it was 22% in case of the kNN and only 11% in case of the DNN.

Liu et al. introduced BeamMaP in [60], which was later improved in [61]. BeamMaP uses Gaussian Process Regression (GPR) as a machine learning regression technique on the beamforming transmission patterns. The improved BeamMaP uses adaptive beamforming as a candidate for building the testing process since it can cover a larger area of MUs and provides more comprehensive interference rejection compared to switched beamforming. They argue that the improved BeamMaP not only provides more efficient coverage, but also reduces base station power consumption. The simulations took place in an outdoor environment with LOS and NLOS areas in different weather conditions, and the GPR model was compared with the kNN and SVM models. The improved adaptive BeamMaP shows better performance than the original BeamMaP in different weather conditions, although it achieves comparable performance to other machine learning methods such as kNN and SVM in dynamic environments. The obtained RMSE performances of BeamMaP are proved to be close to the Bayesian Cramer–Rao bounds. The proposed method outperformed the other models and achieved an MAE of 3.5 m at 1 dB shadowing noise in the simulated dynamic environment. BeamMaP outperformed the kNN and SVM models even at higher noise levels, but its accuracy decreased significantly as the noise level increased.

El Boudani et al. [64] proposed DNN models (one for horizontal and one for vertical positioning) for RSS fingerprint-based 3D positioning in a 5G IoT setup testbed. They compared the results of the DNN model with the performance of SVM and KNN models. In 2D (horizontal) positioning, the DNN model showed the best accuracy with a 1.6 m mean error. For the vertical positioning, the authors trained the models to classify the samples into three classes of height (0.25 m, 0.75 m, and 1.75 m). In this case also, the DNN achieved the best results by perfectly classifying the samples in the 1.75 m class, and more than 95% of the samples were accurately classified in the other two class.

Prasad et al. [62,65] proposed a supervised ML approach based on Gaussian Process (GP) regression to position users in a distributed massive MIMO system using uplink RSRP. They focused on the scenario where noise-free RSS is available for training but only noisy RSS is available for estimating the user’s location. The authors consider the Conventional GP (CGP) and Numerical approximation GP (NaGP) in [65], while in [62], they use the Gaussian approximation GP (GaGP) and the reconstruction-cum-Gaussian approximation GP (RecGaGP) method. It is shown that the achieved RMSE of all GP is close to the Bayesian Cramer–Rao lower bound. In [65], the methods are compared to three baseline methods, which are as follows: the linear least squares, improved linear least-squares trilateration schemes, and the kNN. Both GP methods outperformed the baseline methods and achieved almost the same error in LOS and NLOS indoor scenarios. In [62], the GaGP and RecGaGP methods are compared to the NaGP and CGP methods in outdoor scenarios. The GaGP method had similar results as the CGP and NaGP methods but the RecGaGP outperformed all of them by a large margin.

Huang et al. [66] proposed the following pipeline for indoor positioning. Firstly, they used a Kalman filter to preprocess the raw RSS values. Then, a universal Kiring algorithm is utilized in order to improve the resolution of the fingerprint database. Ultimately, a kNN model is used to estimate the position of the UE. Their experiments were performed in two indoor office rooms. In each room, one 5G NR BS was deployed 3.62 m high above the floor. The data for testing were collected in multiple unconnected test points. According to their results, in the first room, the Kalman filter improved the performance by 31% and the universal Kiring enhanced the accuracy by an additional 26%. In case of the other room, the Kalman filter increased the performance by 6% and the universal Kiring with 36%. Due to the improvements listed above, the achieved error was less than 1.6 m in more than 80% of the test samples.

Gao et al. [67] proposed a dataset generating method, whose output is suitable for 5G high-precision localization. This includes the multilevel feature synthesis method, which flattens the features of a multiple-input multiple-output channel into a single image in order to improve the information density and the robustness to noise. They also introduced the multipath res-inception, which is a deep learning solution for positioning. In this method, in order to extract position related features in the frequency domain and capture the fine channel differences between adjacent antennas, multiple specially sized filters are created. Their simulations were executed in an indoor scenario, and the positioning results show a 0.28 m mean error and 90% of the samples have an error less than 0.51 m. Moreover, in an urban canyon environment they achieved a 0.204 m mean error and less than 0.36 m for 90% of the samples. According to their comparison, the proposed method outperformed the results of four other positioning solutions (TDoA, AoA, kNN, and TDoA-AoA) in both environments.

### 4.2. Lessons Learned

The presented papers show how the emerging communication technologies (e.g., massive MIMO, mmWave communication) of 5G networks can be used for positioning with machine learning. These new technologies increase the amount of data and the number of features available for positioning. Most of these new features (e.g., AoD) can be handled with traditional techniques (e.g., triangulation), but the combination of these features and the increased amount of data make the ML techniques easily manageable compared to traditional techniques. The main advantage of ML algorithms is rapid model development and updating, as new data and measurement types can be used and combined immediately. This makes them particularly well suited as a replacement for traditional fingerprinting algorithms, especially in cases where measurements have a complex relationship to positions.

Results show that in ideal scenarios (low noise, high number of antennas or BSs, LOS propagation), a MAE of less than 2 m can be achieved in indoor and outdoor environments. In NLOS scenarios and high noise scenarios, the MAE becomes much higher and can easily exceed 20 m. It should be noted that in most cases, positioning methods are tested in a fully or partially simulated environment, due to the lack of deployed 5G networks. Simulations tend to underestimate the effects of the physical environment, as many of these effects are difficult to estimate and depend heavily on the particular environment. Models vary greatly in several parameters, such as the number of LoS base stations, the type of simulated signal, and the size of the virtual space; therefore, a comparison of these positioning solutions based only on their positioning error could be very misleading.

## 5. Beyond 5G

The evolution of expectations, and thus solutions, in cellular positioning remains unbroken—just as the emergence of commercial use cases such as factory automation, transportation, and logistics have shaped 5G positioning, advanced commercial applications of the future such as extended reality (XR) gaming, telemedicine, driverless vehicles, and autonomous industrial systems will largely influence the evolution of cellular networks beyond 5G [25,69,70,71,72,73].

**Figure 6:**
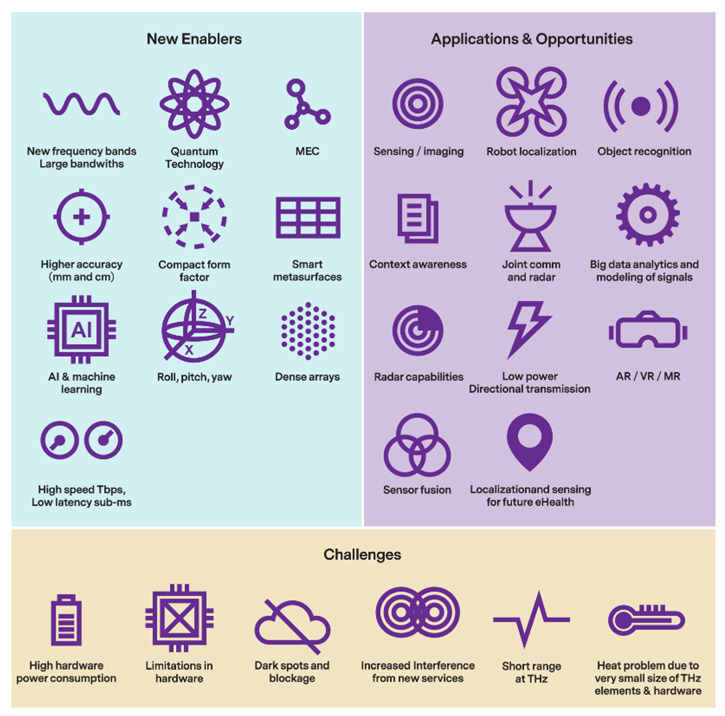
The enablers, new applications, and challenges of 6G [69].

The 6G network will aim to satisfy the challenging requirements of these new applications based on its unprecedented technological advancements. These advancements include even higher frequency ranges, wider bandwidths, massive antenna arrays, intelligent surfaces, intelligent beam-space processing, AI and machine-learning-based techniques, sidelink solutions, architecture evolution, and beyond connectivity [25,69,70,71,72,73,74,75]. These new technologies (as illustrated by Figure 6) will enhance legacy solutions through efficiency and cost optimization [25,74], and open up new possibilities for 6G localization [25,69,70,71,72,73].

In order to satisfy the requirements of new technologies, networks beyond 5G will be forced to conform to even higher standards related to positioning, translating to possibly under 0.5 ms latency, up to eight nines reliability and down to centimeter-level positioning accuracy [72,75,76], or even sub-centimeter level relative-positioning accuracy [73]. The possible capabilities of 6G networks as opposed to 5G are summarized in Table 3.

In current 5G NR positioning, position estimation is based on three components: signal strength, which is often inaccurate because both the path loss of the radio channel and the exact gain of the transmitter and receiver chains are unknown, time-of-arrival, which is limited by the bandwidth of the Positioning Reference Signal (PRS), or angle measurements whose accuracy is limited by the size of the antenna array or the aperture [25].

Possible solutions to overcome these accuracy limitations on the way to 6G are carrier aggregation for positioning and carrier-phase based positioning [77]. Carrier aggregation aims to achieve a higher overall effective bandwidth by combining PRSs received on different carriers into one signal with a very high overall bandwidth [25]. Carrier-phase-based positioning methods [25] use a different approach in which the phase of the correlator output signal varies quickly with increasing propagation distance. One full phase cycle corresponds to one wavelength of the carrier signal. Since the carrier frequency is usually at least a factor of ten or more higher than the bandwidth, the achievable accuracy is about this factor higher, assuming that it is possible to measure a certain fraction of a full phase cycle [25]. The same principle is used in GNSS-RTK (Global Navigation Satellite System-Real Time Kinematic) to achieve centimeter accuracy [25]. All phase measurements need to be performed on the Line-Of-Sight (LoS) path—this requires advanced LoS/NLoS (Non-LoS) detection and separation methods. Standard support to identify NLoS paths and mitigate their impact on timing measurements is already part of the 17th 3GPP release, but this needs to be extended to phase measurements and higher accuracies [25].

Aside from accuracy, low latency is another critical factor for delivering effective positioning services in 5G and beyond [70]. The applications strongly related to positioning in 5G networks and beyond are associated with an increased level of automation, which requires the location of objects (such as automated vehicles) to be obtained at a millisecond level [25,70,74]. New latency solutions being considered for 6G include: allowing inter-layer interactions at the Radio Access Network (such inter-layer interactions would allow the RAN nodes to directly access LTE positioning protocol messages, which could drastically reduce the overall latency budget of positioning sessions), shortening the distance between the location server and the NG-RAN (which could be crucial for latency-intolerant 6G applications), and UE-based positioning, which could transform cellular positioning toward UE-based calculation in a network-controlled manner [25].

Additionally, artificial intelligence and machine learning are expected to play an important role in the cellular network of the future [25,69,70,71,73], especially in conjunction with traditional positioning solutions. AI is expected to have an impact on mobile positioning for Non-Line-Of-Sight (NLoS) multi-paths and possibly on achieving ultra-low latency [70]. It is predicted to be especially crucial in data-rich and complex localization applications (e.g., poor GNSS channel conditions indoor and outdoor urban environments) where we have a large number of multi-modal, indirect, and noisy observations and the physical properties of the system’s nonlinear signal characteristics may be unknown or difficult to model [69]. Another application of AI and ML methods could be semantic localization, as suggested in [73]. Semantic localization means that the position of the target is not given with numerical coordinates, but with a semantic description, such as “put this equipment on the table or in the recycle bin”. In this case, the AI algorithm needs to interpret the connection between the physical coordinates and semantic locations.

Possible further novelties expected in networks beyond 5G include lower instantaneous PRS bandwidth for positioning (to reduce complexity), sidelink solutions to enable localization in areas with partial coverage or no cellular coverage, decentralized architecture, device-free localization, the inclusion of location functionalities in the RAN, as well as intelligent environments and Intelligent Reflective Surfaces (IRS) [25,70,72].

Among these, a lot of attention has recently been given to intelligent reflecting surfaces in particular, which comprise an array of IRS units, each of which can independently incur some change to the incident signal (such as amplitude, frequency, or even polarization, but most often a phase shift) [72,78]. IRS are meant to intelligently configure the wireless environment to help the transmissions between the sender and receiver in case direct communications have bad qualities [78], and they offer a cost-effective solution to link blockage problems in mmWave communications [79]. In [79], a method using random beamforming and maximum likelihood estimation to calculate the AoA and AoD of the line-of-sight path between BS/AP (or IRSs) and the mobile terminal is shown, which then, with the estimated AoDs, proposes an iterative positioning algorithm that achieves centimeter-level positioning accuracy. In [80], a systematic overview of existing works is presented on IRS/RIS, mainly from the signal processing point of view, by focusing on channel estimation, transmission design, and radio localization issues.

Similarly, edge intelligence—a novel technological framework focusing on the seamless integration of AI, communication networks, and mobile edge computing—is widely recognized to be one of the most sought after functions for wireless 6G cellular systems [81]. It conventionally consists of sensing, communication, training, and inference stages, where sensing and communication are executed sequentially, often leading to an excessive amount of dataset generation and uploading time [82]; however, novel solutions, such as integrated sensing and communication (ISAC) introduced in [73,82], merge the sensing and communication stages in order to make the best use of the wireless signals for the dual purpose of dataset generation and uploading. In addition, to address the additional interference between sensing and communication functionalities that ISAC introduces, this paper also proposes a classification error minimization formulation to design the ISAC beamforming and time allocation [82].

Recently, increasing attention has been paid to device-free localization [83,84,85], i.e., the ability to detect and track objects that do not communicate with the localization infrastructure or do not want to be detected and localized at all. These technologies rely on signals designed for target detection and localization (active radar) or signals emitted by other sources of opportunity (passive radar) that are used for localization. Unlike UE localization, device-free localization can use any modulated signal at any operating frequency. As the wireless industry moves toward frequencies above 90 GHz (and eventually Terahertz frequencies) in the future, several Gigahertz wide frequency ranges will become available. Difficult indoor conditions (e.g., multipath effects and signal obstructions) can be mitigated by using waveforms characterized by this wide bandwidth, e.g., UWB waves, using prior knowledge of the environment, selecting reliable measurements, and employing various signal processing techniques [83,85]. UWB technology provides exceptional resolution and localization accuracy in harsh environments because of its ability to resolve multipath effects and penetrate obstacles. These characteristics have helped make UWB an ideal candidate for non-collaborative object detection in short-range radar sensor networks. Device-free localization could be used for fall detection in assisted living facilities. Security applications such as intruder detection in offices are enabled by device-free localization without the knowledge or cooperation of the intruder [85].

Another key technology prominent for 6G network applications such as intelligent transportation systems is data-driven wireless sensing, such as Human Motion Recognition (HMR). HMR systems currently use Support Vector Machines (SVMs) and Convolutional Neural Networks (CNNs) to classify radar signals, but new solutions such as the Deep Spectrogram Network (DSN) introduced in [86] are paving the way for the technology with a significant reductions of recognition errors.

Clearly, numerous challenges await on the road to 6G, particularly tied to the evolution and integration of machine learning and artificial intelligence, which will be expected from networks beyond 5G. The development of intuitive AI solutions without excessive storage needs and their integration into 6G networks without compromising low latency will be one of the key challenges while building the network of the future.

Given that the driving applications in 6G (AR/VR/XR gaming, low-cost tracking and new industrial applications) have very different, and sometimes even conflicting-requirements, they will also require flexible and scalable positioning solutions and location service architectures, which will undoubtedly be expected from the next-generation cellular network.

## 6. Use-Case Examples

Positioning of devices in indoor environments was a focus area of 3GPP Release 16 [87], and further enhancements are expected in Release 17 [88]. With the fast advances of 5G standardization, several new indoor positioning use cases are brought along. They fall into the following categories:

1 Location-based services-related use cases include AR- and VR-scenarios, telepresence and wearables for entertainment [89] and educational [90] reasons, as well as smart advertising [91], navigation, building occupancy-count estimation [92], and social networking [14].
2 Industrial use cases include trolley location, waste management, container handling, manufacturing, warehousing, and Industrial Internet of Things (IIoT) [88,93,94,95].
3 eHealth-related use cases are split to positioning of people and medical equipment, including patient location, remote health care, and remote surgery [96,97,98].
4 Emergency and mission critical use cases are related to emergency services, first responders, alerting nearby responders, emergency vehicle and equipment location [14].
5 Road-related use cases include traffic monitoring, management and control, V2X, car and bike sharing, as well as flow control in transportation hubs and public transportation [99].
6 Rail, maritime, and aerial use cases are related to asset tracking (wagon and container), drones (transport and inspection), mining, and underwater and aerial unmanned vehicles [100,101,102,103].

Expectations for 5G- and 6G-based positioning depend on the application area. A comprehensive summary of positioning requirements for different use cases can be found in [87]. This standard defines positioning requirements, such as horizontal and vertical accuracy, availability, heading, and latency for some typical scenarios, given that the UE moves slower than a given speed.

### 6.1. Positioning in Industrial Settings and Cyber–Physical Systems

When it comes to certain use cases, general process automation tasks require sub-meter accuracy, where the latency of positioning estimation is <2 s. A very different use case is inbound logistics for manufacturing, where the requirement on both horizontal and vertical accuracy should be below 20 cm with a positioning estimation latency of <1 s. Regarding this latter parameter, augmented reality applications for smart factories have much stricter latency criteria: less than 15 ms. Although the standard discusses various other use cases as well, their requirements fall within the range of the previously listed scenarios [88].

Industrial use cases have extraordinary potential to benefit most from the positioning capabilities of 5G and 6G networks. Position information can help optimize and automate processes in various vertical sectors, from logistics and manufacturing to mining and transportation [2]. In industrial control and factory automation, location information is of great benefit to both sides of communication: to the (mobile) terminals (or robots) to perform their tasks, and to the network to allocate and control resources and increase processing efficiency.

The requirements of cyber–physical control applications (i.e., in factories of the future) are covered by the 3GPP standard [88]. Here, the main categories of generic 5G-related use cases are (i) factory automation, (ii) process automation, (iii) HMIs and production IT, (iv) logistics and warehousing, and (v) monitoring and maintenance. Among them, logistics and warehousing are the prominent case for indoor positioning.

Asset tracking is a trivial use case, not only in warehousing [93], but also in smart product manufacturing [94] and smart logistics [95]. In all of these scenarios, 5G and 6G positioning comes into play, linking location data to the status change of the tracked asset. For smart products, not only the change in physical position is logged and time-stamped, but the change in various environmental variables (e.g., temperature, vibration, acceleration during carriage, etc.) could also be noted in the asset’s history.

When it comes to service guarantees, the 5G slice for massive Machine Type Communication (mMTC) would be best suited for information exchange in these use cases [104]. As for warehousing technology, indoor logistics are operated through AGVs or forklifts, and in less advanced settings, human-operated, manual (engineless) carts. In either case, the moving logistics equipment is the one to track, and the products themselves could be linked to the equipment upon pickup (e.g., through RFID) so that their location can be tracked even while in motion [93]. Nevertheless, both indoor and outdoor logistics require large amounts of interaction with the public network.

### 6.2. Specialties of V2X Positioning

Of all the use cases related to 5G positioning, V2X scenarios appear most frequently in scientific publications and reports. This is mainly due to the increased public expectations for autonomous vehicles on public roads. In general, 5G V2X requirements (in terms of throughput, delay, and other QoS metrics) are set in 3GPP recommendation TS22.186 [105] for various use cases. These include trajectory sharing and coordinated driving, vehicle platooning, and remote driving, among others, which may or may not require position data retrieval and sharing. Begheri et al. [106] have summarized the key features and roadmap of the new 5G wireless system that will help meet these requirements. Similar to other authors, they emphasize the importance of sensor fusion, multi-connectivity, and the various security aspects of 5G V2X communications. They also devote a short chapter to precise positioning and describe the main features of NR-V2X that contribute to more precise positioning compared to LTE-V2X.

According to Wymeersch et al., positioning for V2X applications is supported by five properties of 5G (and later 6G): high carrier frequencies, large bandwidths, large antenna arrays, device-to-device communications, and ultra-dense networking [107]. They reinforce the general view that accurate positioning will rely on a combination of sensors. In the highlighted promising research directions, gaps identified include tracking algorithms, information fusion of device-centric and network-centric positioning, accuracy, and reliability.

Bartoletti and her co-authors focus on how 5G and beyond positioning can support vehicle safety applications [108]. They emphasize that joint communication strategies, such as integrated mobile communication, positioning, and radar (in short: JRC ), will be key to safety-critical vehicular applications. One of their concluding remarks is that during the definition and standardization of 6G, the communication parameters that affect JRC performance should be watched closely. Another important suggestion is that the waveform design (such as OFDM) should consider radar performance requirements for vehicular safety scenarios.

In their paper, Fouda et al. analyze various solutions for long-term and high-precision positioning for new V2X mobile radios [109]. They propose a novel selection-positioning method to dynamically switch between GNSS and downlink TDOA measurements based on the locations of V2X UEs and the accuracy of the collected measurements.

## 7. Conclusions

In this paper, we provided an overview of the positioning approaches, methods, and algorithms that are envisioned and used with the help of 5G and 6G mobile networks. The main goal of the paper was to provide a comprehensive overview of Machine Learning (ML) aided positioning techniques; however, in order to introduce those, the positioning approaches used in cellular networks had to be summarized first. After describing the state-of-the-art techniques and enhanced algorithms, we surveyed the published use-case results in various application areas—especially for industrial cyber–physical systems and the V2X domain.

Machine learning plays an important role in the control and optimization of 5G and beyond networks. Furthermore, ML solutions improve various cellular-based solutions, including positioning. Regarding 6G-supported indoor and outdoor positioning, several new approaches are being investigated, including inter-layer interactions at the Next Generation Radio Access Network (NG-RAN), as well as the extensive usage of mobile edge computing, where the location servers are closer to the NG-RAN. Although 6G standardization has just started, it is clear that there are numerous new solutions for latency control. These include allowing inter-layer interactions at the Radio Access Network, shortening the distance between the location server and the NG-RAN, and UE-based positioning. Mixing UE-based positioning with cellular-based positioning is also an interesting approach, where the UE-based calculations are supported with network-controlled measurements.

Aside from aiming to be an extensive study of the cellular-based positioning domain, the additional value of the paper lies in its comparison tables, where the various approaches, methods, and results are compared for both the conventional solutions and those enhanced with machine learning capabilities. The subsections on lessons learned also helps the reader find the core messages and the most promising solutions for enhanced positing that are based on 5G and 6G network capabilities.

## Figures and Tables

**Table 1:** Comparison of the conventional positioning techniques in 5G networks.

Refs.	Algorithm	Input Data Type	Simulation?	Environment	Error
[33]	Robust Weighted Least Squares + RANSAC and IDD combined	TDoA	realistic	indoor	<3 m
[34]	Dynamic reconstruction fingerprint matching algorithm	Received signal strength indicator	simulated	indoor	<1 m
[35]	Extended Kalman filter	AoA	realistic	outdoor	<1 m
[36,37]	Extended Kalman filter	Uplink reference signal	simulated	outdoor	<1 m
[39]	Expectation maximization, subspace-spaced algorithm	Uplink reference signal	simulated	indoor	<1 m
[40]	Unscented Kalman filter	AoA, ToA	simulated	indoor	<1 m
[41]	Deriving Cramer–Rao bound	AoA, TDoA	realistic	outdoor (vehicle)	<1 m
[43]	Taylor series least-square method	GNSS-TOA, 5G-AoA	simulated	outdoor	<10 m (95%)
[44]	Deriving Ficher information of 5G and GNSS signals	Simulated GNSS, simulated 5G signals	simulated	outdoor	<1 m
[45]	Particle filter	Real GNSS, simulated 5G signals	simulated	outdoor	<3 m (RMSE)
[46]	OFDMA-based VLCP	Light signals, RSS	simulated	indoor	<1 m

**Table 2:** Comparison of the machine-learning-aided positioning techniques in 5G networks.

Refs.	ML Method	Measurement Type	Simulation/ Realistic	Environment	Error
[49]	NN, RF	BRSRP	realistic	outdoor	<10 m (80%)
[53]	kNN, ELM	CSI	realistic	outdoor	8.2m
[54]	NN, TDNN (time-delay neural network)	TOA, code phase estimate	realistic	outdoor	4.9 m (ranging RMSE)
[55]	NN	AoA	hybrid	both	0.4 m
[56]	Densely connected Neural Network	RSS, GNSS signal	simulation	outdoor	0.74 m
[57]	NN, DT	BRSRP	simulation	outdoor	1.4 m
[50]	CNN, LSTM, TCN	Beamformed fingerprint	simulation	outdoor	1.78 m
[58]	weighted kNN	CSI	simulation	outdoor	2 m (90%)
[59]	Deep convolutional Gaussian process	Beamforming images	simulation	outdoor	2.79 m
[27]	13 ML models including NN, kNN, RF	RSRP	simulation	outdoor	3.3 m (kNN)
[60,61]	GPR, kNN, SVM	RSRP	simulation	outdoor	3.5 m
[62]	Gaussian Processes	RSRP	simulation	outdoor	10 m
[63]	NN, kNN, SVM	RSRP	simulation	indoor	1.6 m
[64]	DNN	RSS	simulation	indoor	1.6 m
[65]	Gaussian Processes	RSRP	simulation	indoor	<2 m
[66]	kNN	RSS	realistic	indoor	<2 m
[67]	NN	CSI	simulation	both	<1 m

**Table 3:** The possible capabilities of 6G as opposed to 5G [74].

Major Factors	6G	5G
Peak data rate	>100 Gb/s	10[20] Gb/s
User experience data rate	>10 Gb/s	1 Gb/s
Traffic density	>100 Tb/s/km^2^	10 Tb/s/km^2^
Connection density	>10 million/km^2^	1 million/km^2^
Delay	<1 ms	ms level
Mobility	>1000 km/h	350 km/h
Spectrum efficiency	>3x relative to 5G	3–5x relative to 4G
Energy efficiency	>10x relative to 5G	1000x relative to 4G
Coverage percent	>99%	∼70%
Reliability	>99.999%	∼99.9%
Positioning precision	Centimeter level	Meter level
Receiver sensitivity	<–130 dBm	About –120 dBm
